# Mechanism and rational combinations with GP‐2250, a novel oxathiazine derivative, in ovarian cancer

**DOI:** 10.1002/cam4.70031

**Published:** 2024-08-08

**Authors:** Mark S. Kim, Deanna Glassman, Katelyn F. Handley, Adrian Lankenau Ahumada, Nicholas B. Jennings, Emine Bayraktar, Katherine Foster, Robiya Joseph, Sanghoon Lee, Robert L. Coleman, Anil K. Sood

**Affiliations:** ^1^ Department of Gynecologic Oncology and Reproductive Medicine The University of Texas MD Anderson Cancer Center Houston Texas USA; ^2^ Department of Gynecologic Oncology H. Lee Moffitt Cancer Center and Research Institute Tampa Florida USA; ^3^ Division of Gynecologic Oncology, Department of Obstetrics and Gynecology, Morsani College of Medicine University of South Florida Tampa Florida USA; ^4^ MD Anderson Cancer Center UTHealth Graduate School of Biomedical Sciences Houston Houston Texas USA; ^5^ US Oncology Research The Woodlands Texas USA

**Keywords:** anti‐neoplastic drug, bevacizumab, GP‐2250, ovarian cancer, PARP inhibitor

## Abstract

**Background:**

GP‐2250, a novel analog of taurultam (TRLT), has emerged as a potent anti‐neoplastic drug; however, the mechanisms underlying its effects are not well understood. Here, we investigated the mechanism of action and the biological effects of GP‐2250 using in vitro and in vivo models.

**Methods:**

We carried out a series of in vitro (MTT assay, Annexin V/PI assay, colony formation assay, reverse‐phase protein array [RPPA], and HRLC/IC analysis) to determine the biological activity of GP‐2250 and investigate the mechanism of action. In vivo experiments were carried out to determine the therapeutic efficacy of GP‐2250 alone and in combination with standard‐of‐care drugs (e.g., paclitaxel, cisplatin, topotecan, and poly ADP‐ribose polymerase [PARP] inhibitors).

**Results:**

We investigated the cytotoxic effect of GP‐2250 in 10 ovarian cancer cell lines and found GP‐2250 combined with a PARP inhibitor had the greatest synergy. RPPA revealed that GP‐2250 inhibited hypoxia‐inducible factor‐1α, AKT, and mammalian target of rapamycin (mTOR) activation and expression. High‐resolution mass spectrometry revealed that hexokinase2 activity and protein expression were significantly reduced by GP‐2250 exposure. Furthermore, GP‐2250 reduced glycolysis and ATP synthesis in cancer cells. An in vivo pharmacodynamic experiment using the OVCAR8 mouse model demonstrated that 500 mg/kg GP‐2250 was effective in downregulating AKT and mTOR activation and expression. In the in vivo therapy experiment using an orthotopic mouse model, a combination of GP‐2250 with either PARP inhibitors or bevacizumab showed a significant reduction of tumor weights and nodules compared to those treated with a vehicle, control IgG groups, or monotherapy groups.

**Conclusions:**

Taken together, our data indicate that GP‐2250 exerts profound effects on tumor metabolism and, in combination with PARP inhibitors or bevacizumab, showed promising anti‐tumor efficacy. These findings could have implications for the clinical development of GP‐2250.

## 1 INTRODUCTION

**FIGURE 1:**
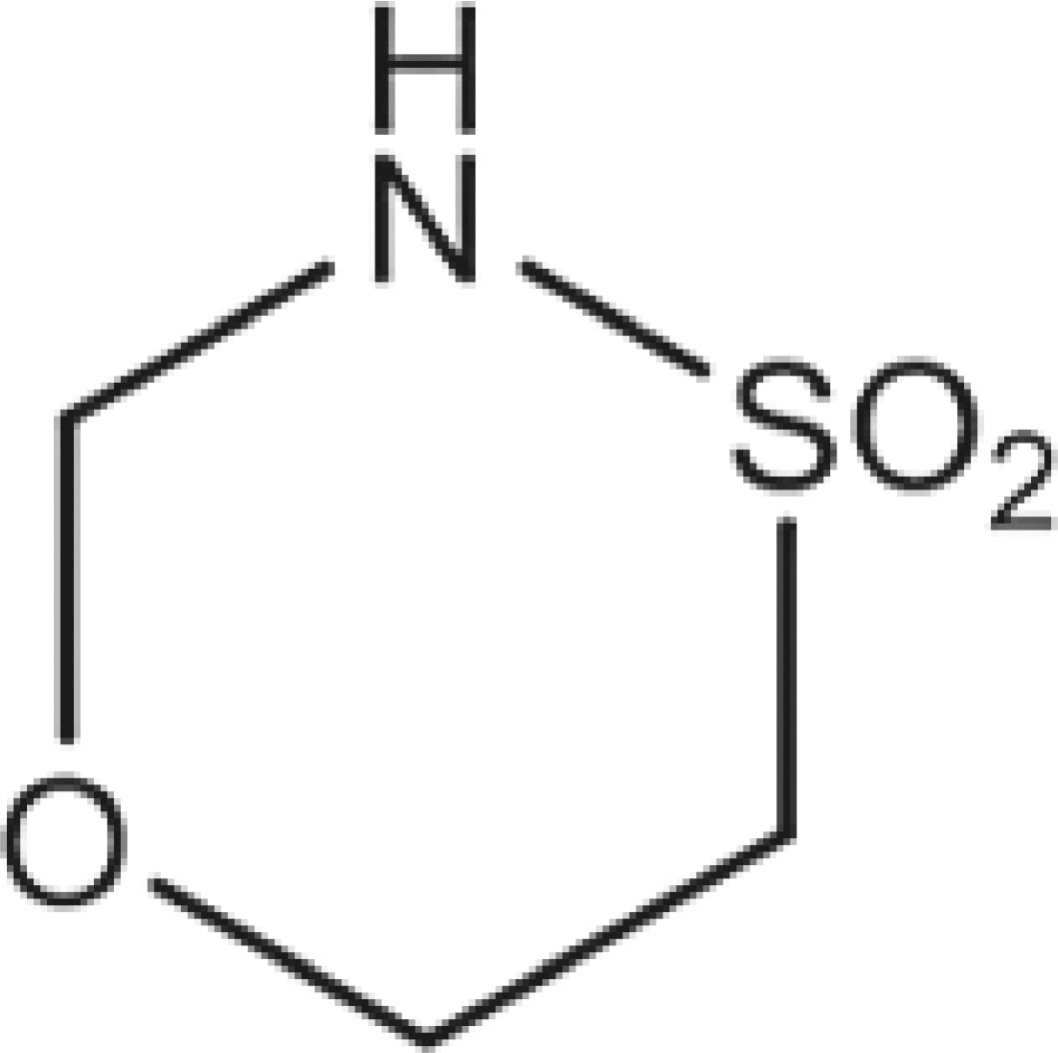
Molecular structure of GP‐2250 (1.4.5‐oxathiazan‐dioxid‐4.4), an oxathiazine derivative of taurultam with a molecular weight of 137.5 g/mol. The chemical structure of GP‐2250 presented in Figure 1 has been reproduced from the study by Buchholz M et al. [15]. In compliance with *BMC Cancer* guidelines, this figure may be used without restriction, provided it is properly cited. There is no requirement to obtain permission to use this figure.

Ovarian cancer remains the leading cause of death among all gynecological cancers.^1^, ^2^ Almost 70% of ovarian cancer patients present with advanced disease at diagnosis, and most patients die of relapsed disease by 5 years after diagnosis. The best standard therapeutic regimen for advanced ovarian cancer is cytoreductive surgery and systemic chemotherapy. Maintenance therapy with bevacizumab, an anti‐vascular endothelial growth factor (VEGF) antibody (AVA), or a poly (ADP‐ribose) polymerase (PARP) inhibitor is increasingly used following upfront therapy.^3^ About half of ovarian cancer cases have homologous recombination DNA repair defects in response to double‐strand breaks.^4^ The role of PARP inhibitors in treating homologous recombination‐deficiency (HRD) ovarian cancer has been well studied. However, the benefits for those with homologous recombination proficient (HRP) ovarian cancer are limited.^5^, ^6^ Thus, new therapies are needed to improve the clinical outcomes of patients with ovarian cancer. Taurolidine (TRD), a substance derived from the amino acid taurine, has been clinically used to prevent catheter‐related bloodstream infections.^7^ TRD has antiproliferative and antineoplastic activity in vitro and in vivo against various cancer types, such as glioblastoma,^8^ melanoma,^9^ mesothelioma,^10^ and colon carcinoma,^11^ with minimal to no toxicity in patients with gastric carcinoma or glioblastoma.^12^, ^13^ The favorable safety profile of TRD makes it a promising agent for novel application in cancer treatment. However, TRD use is limited owing to a short half‐life. Researchers recently developed the oxathiazinane derivative GP‐2250 (1,4,5‐oxathiazan‐dioxide‐4,4) (Figure 1). They demonstrated that it has an increased metabolic half‐life and antineoplastic effects on pancreatic tumor cells as well as patient‐derived xenograft models of pancreatic adenocarcinoma.^14^, ^15^ Therefore, we investigated the biological effects of GP‐2250 in combination with standard‐of‐care drugs on ovarian cancer models. As described herein, we evaluated the therapeutic effect of GP‐2250 in combination with the PARP inhibitors olaparib, niraparib, and rucaparib and with bevacizumab in ovarian cancer models in vivo and in vitro. Based on our results, we hypothesize that GP‐2250 has antineoplastic effects on ovarian cancer, especially in combination with a PARP inhibitor or bevacizumab. Our findings provide evidence for the clinical development and use of these combinations in patients with ovarian cancer.

## 2 MATERIALS AND METHODS

### 2.1 Cell lines and culture conditions

The human ovarian cancer cell lines A2780, Caov3, HeyA8, HeyA8‐MDR, Kuramochi, OVCAR3, OVCAR4, OVCAR5, OVCAR8, and SKOV3 were obtained from the ATCC and University of Texas MD Anderson Cancer Center Cytogenetics and Cell Authentication Core. All cells were cultured in RPMI 1640 medium (HyClone) supplemented with 10% fetal bovine serum (Sigma‐Aldrich) and 1% gentamycin at 37°C with 5% CO_2_ and ambient atmospheric O_2_. The cells were cultured with 1% O_2_ as indicated for a hypoxia experiment. All cell lines were authenticated by the MD Anderson Cytogenetics and Cell Authentication Core using short tandem repeat fingerprinting. Also, they were tested for mycoplasma contamination using polymerase chain reaction (PCR). Cells were used within 20 passages after thawing for in vitro experiments and 10 passages after thawing for in vivo experiments.

### 2.2 Reagents

GP‐2250 powder (Geistlich Pharma) was set to physiological pH after being dissolved in Lactated Ringer's solution (Avantor) and subsequently sterile‐filtered. The preparation was freshly performed and used within 2 h.

### 2.3 Cell viability assay

Cell viability assays were performed to evaluate the cytotoxic effects of GP‐2250 and standard chemotherapy drugs (paclitaxel, cisplatin, topotecan, and olaparib) alone and in combination. Ovarian cancer cells were seeded in a 96‐well plate at a density of 3000 cells per well in a 100 μL total volume with quadruplicate replicates. After cells were incubated for 24 h, the culture medium was removed and replaced with a medium containing serial dilutions of GP‐2250 and chemotherapy drugs. The cells cultured with cisplatin were incubated for 96 h, and those cultured with the other drugs were incubated for 72 or 96 h. Cell viability was determined using a CellTiter‐Glo Luminescent Cell Viability Assay (Promega) according to the manufacturer's instructions. The viable cell luminescence was quantified using a Multilabel Plate Reader (Agilent, Cytation 5). Dose–response curves were plotted using Prism software (version 9.0.0; GraphPad Software). The CompuSyn software program (http://www.combosyn.com/) was used to examine drug–drug interactions in the results of fraction affected‐combination index (CI) plots. A CI of less than 1.0 indicated a synergistic effect, whereas a CI greater than 1.0 indicated an antagonistic one.^16^ All experiments were performed in triplicate.

### 2.4 Western blotting

Total protein cell lysates were extracted using a modified RIPA buffer (1% Triton X‐100, 25 mmol/L Tris, pH 7.4, 150 mmol/L NaCl, 0.1% sodium dodecyl sulfate, 0.5% sodium deoxycholate) with protease and phosphatase inhibitors (Roche). BCA Protein Assay Reagent (Thermo Fisher Scientific) was then used to measure protein concentrations in the lysates. Equal amounts of isolated proteins were separated via electrophoresis on 4%–12% NuPAGE gels (Thermo Fisher Scientific) and transferred to nitrocellulose membranes. After blocking with a Tris‐buffered saline solution with 0.1% Tween 20 containing 5% nonfat milk, the membranes were incubated with primary antibodies (1:1000) at 4°C overnight. The membranes were then exposed to a horseradish peroxidase‐conjugated secondary antibody (1:3000) and visualized using an enhanced chemiluminescence detection kit (Pierce Biotechnology). Anti‐β‐actin, Glyceraldehyde 3‐phosphate dehydrogenase (GAPDH), and vinculin antibody (0.1 μg/mL; Sigma‐Aldrich) were used as a loading control.

### 2.5 Quantitative reverse transcription‐PCR analysis

Briefly, total RNA (1 μg/sample) was extracted from cells using the RNeasy Mini Kit (QIAGEN), and the quantity and quality of RNA were assessed using a NanoDrop spectrophotometer (Thermo Fisher Scientific). cDNA was synthesized using a Verso cDNA Synthesis Kit (Thermo Fisher Scientific) and used as the template in a real‐time PCR assay with Power SYBR Green PCR Master Mix Reagent (Thermo Fisher Scientific) and a 7500 Real‐Time PCR System (Thermo Fisher Scientific). All procedures followed the protocols provided by the manufacturer. The expression of the target hexokinase (HK) genes (*HK1 and HK2*) was calculated using the ΔΔCt method and normalized according to 18S rRNA expression as described previously.^17^ Primers for hexokinases were made by Sigma, with the following primer sequences: HK1 forward primer, CTGCTGGTGAAAATCCGTAGTGG; HK1 reverse primer, GTCAAGAAGTCAGAGATGCAGG; HK2 forward primer, GAGTTTGACCTGGATGTGGTTGC; HK2 reverse primer, CCTCCATGTAGCAGGCATTGCT; 18S forward primer, CGCCGCTAGAGGTGAAATTC; 18S reverse primer, TTGGCAAATGCTTTCGCTC.

### 2.6 Colony formation assay

In the colony formation assay, 2 × 10^3^ cells pretreated with GP‐2250 were seeded into 35 mm culture dishes and further cultured for 7 days. Then, cells were washed using PBS and stained using a CellMAX clonogenic assay kit (BioPioneer) according to the manufacturer's instructions.

### 2.7 RNA interference

Small interfering RNAs (siRNAs) targeting HK1 and HK2 were purchased from Sigma‐Aldrich (sequences are listed in Table S1). Using a Basic Local Assignment Search Tool search, a siRNA with a nonspecific function that did not share sequence homology with any known mRNA was used as a control. Briefly, cancer cells at 50%–60% confluence were transfected with siRNA at a final concentration of 100 nmol/L. Reverse transcription‐PCR and Western blot analysis confirmed knockdown of the HK1 and HK2 proteins after transfection for 48 and 72 h, respectively.

### 2.8 AKT kinase assay

Equal amounts of protein lysates isolated from ovarian tumor samples were used for an AKT kinase assay, and the protein concentration was quantified using the Bradford method. The AKT activity in the lysates was analyzed using an AKT kinase assay kit (Abcam; ab139436) according to the manufacturer's instructions.

### 2.9 HK activity assay

HK activity was determined using a hexokinase activity assay kit (Abcam; ab136957) following the manufacturer's instructions. Cells were incubated for 12 h in a glucose‐free medium (RPMI; Sigma‐Aldrich) prior to the HK activity assay.

### 2.10 Reactive oxygen species assay

A total of 5 × 10^5^ ovarian cancer cells were plated onto 35 mm tissue culture dishes and incubated overnight following treatment with a vehicle or GP‐2250 for 24 h. Reactive oxygen species (ROS) and peroxide levels were measured using a cell‐based fluorogenic assay kit. (Abcam; ab139476).

### 2.11 ATP assay

A total of 2.5 × 10^6^ ovarian cancer cells were incubated overnight onto 100 mm tissue culture dishes. The cells were treated with vehicle or indicated concentration of GP‐2250 for 12 h. Total ATP levels in the cells were then measured using a luminescent ATP detection assay kit (ab113849).

### 2.12 Glycolysis assay

Extracellular lactate was measured using an L‐lactate assay kit (Abcam, ab169557) according to the manufacturer's instructions. Ovarian cancer cells were treated as described above. Treated cancer cells were collected and then homogenized with 110 μL of cold lactate assay buffer on ice and centrifuged at 14,000 *g* for 5 min. The supernatant was used for the lactate measurement.

### 2.13 VEGF ELISA

A total of 2 × 10^6^ ovarian cancer cells were plated into 100 mm tissue culture dishes and incubated overnight at 1% (hypoxic condition) or 20% (normoxic condition). Then, cells were treated with vehicle or GP‐2250 and further incubated for 24 h in either hypoxic or normoxic conditions for the indicated time. The cell culture supernatants were collected and assayed for the secreted level of VEGF according to the manufacturer's instructions.

### 2.14 Immunohistochemical staining

Formalin‐fixed, paraffin‐embedded ovarian tumor sections were stained for AKT, phospho‐AKT, mammalian target of rapamycin (mTOR), and phospho‐mTOR. Paraffin slides were prepared via deparaffinization and antigen retrieval following endogenous peroxidase blocking with 3% hydrogen peroxide and a protein block with 4% fish gelatin. Then, slides were incubated with a primary antibody (1:100 dilution) in 5% goat serum in phosphate‐buffered saline containing 0.1% Tween‐20 (PBS‐T) overnight at 4°C. After incubation with the primary antibodies, the slides were washed with PBS‐T and further incubated with either a peroxidase‐conjugated goat anti‐rabbit or anti‐mouse secondary antibody for 1 h at room temperature. Peroxidase was visualized by incubating with 3,3′‐diaminodbenzidine to monitor for the appropriate staining density. Coverslips were placed on the slides and affixed using a Permount mounting medium (Thermo Fisher Scientific). The images were acquired using a Leica DM4000 Microscope (Wetzlar, Germany), and five mid‐power (20×) microscopic fields per slides were examined using IHC toolbox in the ImageJ software program.

### 2.15 Reverse‐phase protein array (RRPA)

RPPA was performed by harvesting cancer cells treated with vehicle control or GP‐2250 for 24 h and preparing cell lysates using RIPA buffer (1% Triton X‐100, 25 mmol/L Tris, pH 7.4, 150 mmol/L NaCl, 0.1% SDS, 0.5% sodium deoxycholate) containing freshly added protease and phosphatase inhibitors. Protein concentrations were quantified using a BCA Assay Kit (Pierce Biotechnology) and adjusted to 1.5 μg/mL. The RPPA analysis was performed by MD Anderson Cancer Center's Functional Proteomics RPPA Core Facility as described previously.^18^

### 2.16 High‐resolution liquid chromatography/ion chromatography analysis

Cell extracts were prepared and analyzed via ultrahigh‐resolution mass spectrometry to determine the relative abundance of polar metabolites in samples. About 80% of confluent ovarian cancer cells were seeded in 10‐cm dishes in triplicate. Metabolites were extracted using 1 mL of ice‐cold 80/20 (v/v) methanol/water. Extracts were centrifuged at 17,000 *g* for 5 min at 4°C, and supernatants were transferred to clean tubes, which was followed by evaporation of the extracts to dryness under nitrogen. Dried extracts were reconstituted in deionized water, and 5 μL of the extract was injected for ion chromatography‐mass spectrometric analysis. The ion chromatography mobile phase A (weak) was water, and the mobile phase B (MPB; strong) was water containing 100 mM KOH. A Dionex ICS‐5000^+^ system including a Dionex IonPac AS11 column (4‐μm particle size, 250 × 2 mm; Thermo Fisher Scientific) with the column compartment kept at 30°C. The autosampler tray was chilled to 4°C. The mobile phase flow rate was 360 μL/min, and the gradient elution program was as follows: 0–5 min, 1% MPB; 5–25 min, 1%–35% MPB; 25–39 min, 35%–99% MPB; 39–49 min, 99% MPB; 49–50 min, 99%–100% MPB. The total run time was 50 min. For enhanced sensitivity, methanol was delivered using an external pump and combined with the eluent using a low‐dead volume mixing tee. Data were acquired using an Orbitrap Fusion. Tribrid Mass Spectrometer (Thermo Fisher Scientific) in electrospray ionization–negative ionization mode. For hydrophilic interaction liquid chromatography (LC.) analysis, the same samples were diluted in 90/10 acetonitrile/water containing 1% formic acid, and 15 μL following injection for LC‐mass spectrometric analysis. The LC mobile phase A (weak) was acetonitrile containing 1% formic acid, and the LC MPB (strong) was water containing 50 mM ammonium formate. A Vanquish LC system (Thermo Fisher Scientific) with an Intrada amino acid column (3‐μm particle size, 150.0 × 2.1 mm; Imtakt) and a column compartment kept at 30°C. The autosampler tray was chilled to 4°C. The mobile phase flow rate was 300 μL/min, and the gradient elution program was as follows: 0–5 min, 15% MPB; 5–20 min, 15%–30% MPB; 20–30 min, 30%–95% MPB; 30–40 min, 95% MPB; 40–41 min, 95%–15% MPB; 41–50 min, 15% MPB. The total run time was 50 min. Data were acquired using the Orbitrap Fusion Tribrid Mass Spectrometer in electrospray ionization–positive ionization mode at a resolution of 240,000. Raw data files were imported to the TraceFinder software program (Thermo Fisher Scientific) for final analysis. The relative abundance of each metabolite was normalized according to the DNA concentrations.

### 2.17 In vivo models of ovarian cancer

All animal protocols were approved by MD Anderson Institutional Animal Care and Use Committee. Eight‐ to 12‐week‐old female nu/nu mice (*n* = 10) were obtained from Taconic Biosciences. Five mice were housed per cage under pathogen‐free conditions at a constant temperature and humidity. All mice were fed a regular diet and water ad libitum according to American Association for Laboratory Animal Science guidelines and the US Public Health Service Policy on Humane Care and Use of Laboratory Animals. The mice were euthanized via carbon dioxide asphyxiation followed by cervical dislocation once the mice were moribund. To establish xenograft models of ovarian cancer, luciferase‐labeled OVCAR8 ovarian cancer cells were cultured to 70%–90% confluence and then trypsinized, washed twice with phosphate‐buffered saline, and resuspended in ice‐cold Hank's balanced salt solution (CellGro Technologies, 21‐021‐CV). The mice were then inoculated with 1×10^6^ luciferase labeled OVCAR8 cells via intraperitoneal (IP) injection into the right side of the abdomen. Tumor establishment was subsequently confirmed after injection of 200 μL of 14.3 mg/mL luciferin (GoldBio; #LUCK‐1G) using a Xenogen IVIS imaging system (Xenogen). For the pharmacodynamic (PD) study, treatment was initiated 21 days after cancer cell injection once imaging demonstrated tumor establishment. Next, the mice were given treatment twice (24‐h intervals) with one of three dose levels of GP‐2250 (250, 500, or 1000 mg/kg) via IP injection. Then, the mice were euthanized at 6 h, 1 day, and 2 days after treatment. For the therapeutic experiments (*n* = 10), after confirming tumor uptake at 18 days after cell injection, mice were randomly assigned to the following treatment groups: (1) vehicle control, (2) GP‐2250 (500 mg/kg, IP, 3 days a week), (3) PARP inhibitor: olaparib, niraparib, rucaparib, (50 mg/kg, orally, 5 days a week), (4) combination of the GP‐2250 and PARP inhibitor, (5) IgG control (BioX Cell, at 6.25 mg/kg, IP, twice a week), (6) bevacizumab (anti‐human VEGF antibody, at 6.25 mg/kg, IP, twice a week) and (7) combination of GP‐2250 and bevacizumab. PARP inhibitors were reconstituted in 10% dimethyl sulfoxide and 10% 2‐hydroxypropyl‐β‐cyclodextrin (Sigma). Treatment continued for 4 weeks. If any group of mice became moribund, then all of the mice were to be euthanized. None of them were euthanized due to tumor burden. Mouse body weights, tumor weights, and nodule numbers were recorded. Tumor samples obtained from the mice were formalin‐fixed and snap‐frozen for further analysis.

### 2.18 Statistical analysis

All quantitative data were analyzed and compared using Prism software (version 9.0). The Student *t*‐test was used to analyze statistical differences between groups, and *p* values less than 0.05 were considered significant. All statistical tests were two‐sided unless otherwise noted.

## 3 RESULTS

### 3.1 Cytotoxic effect of GP‐2250 on ovarian cancer cells

**FIGURE 2:**
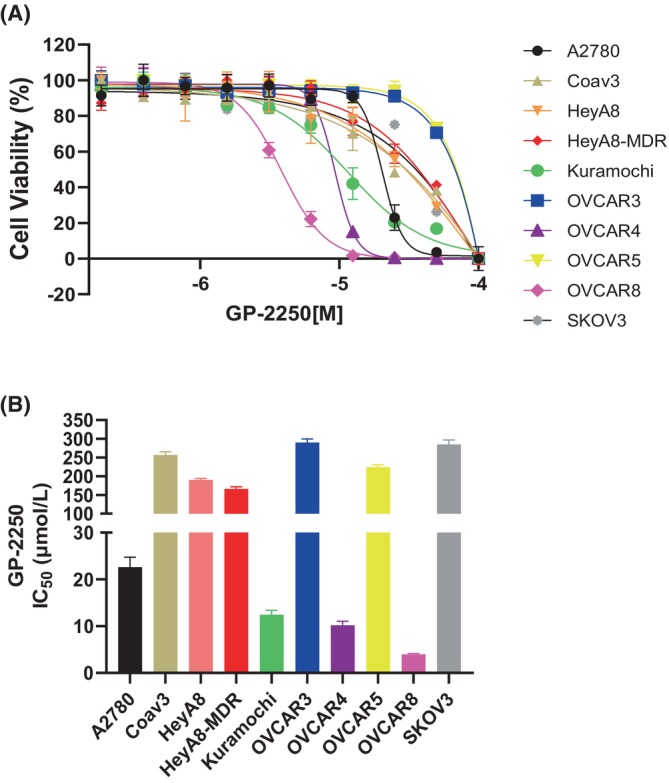
The cytotoxic effects of GP‐2250 on ovarian cancer cells. (A) Cell viability at 72 h after treatment with GP‐2250. The dose–response curves for cell viability are representative of three independent experiments. (B) IC_50_s of GP‐2250. Each IC_50_ was calculated using Prism software (version 9.0). The error bars in the top graph indicate mean (± SD) values and are representative of three biological experiments.

**FIGURE 3:**
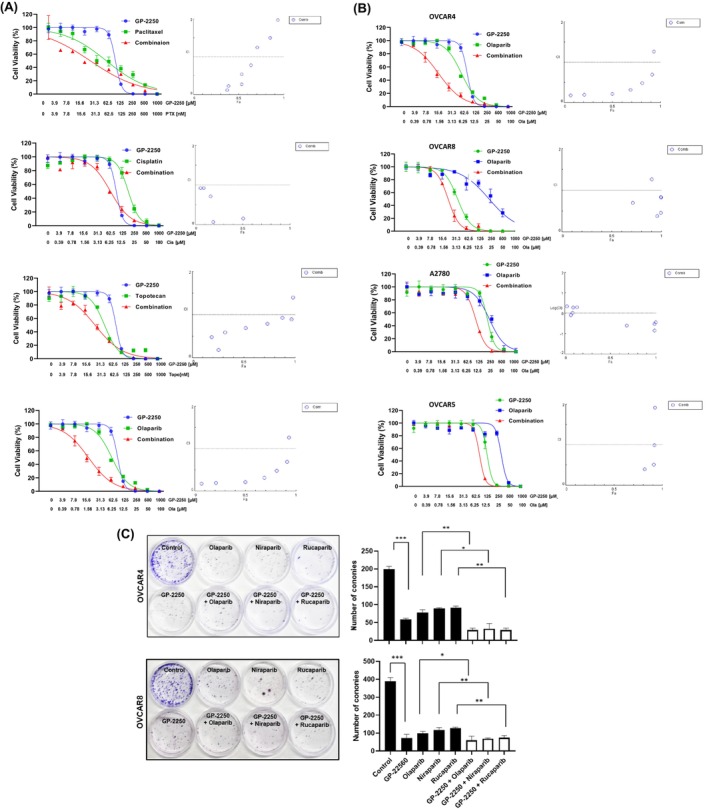
Effects of GP‐2250 and standard‐of‐care chemotherapy drugs on ovarian cancer cells. (A) Cell viability assay. OVCAR4 cells were treated with GP‐2250 and the chemotherapy drugs paclitaxel, cisplatin, topotecan, or olaparib alone and in combinations at the indicated concentrations for 72–96 h. (B) HRD (OVCAR4 and OVCA8) and HRP (A2780 and OVCAR5) ovarian cancer cells were treated with GP‐2250 and the PARP inhibitor, olaparib, alone and in combinations at the indicated concentrations for 72 h. CI values were calculated using CompuSyn software. CI less than 1.0 indicated a synergistic effect, whereas a CI greater than 1.0 indicated antagonism. (C) Colony formation assay. OVACR4 and OVCAR8 ovarian cancer cells (2 × 10^3^ cells per plate) were treated with GP‐2250 and PARP inhibitors alone or in combination and then further cultured for 7 days. The colony was stained using CellMAX clonogenic assay kit. Representative images from three independent experiments are shown. The error bars indicate mean (± SD) values and are representative of three biological experiments. *p < 0.05; **p < 0.01; ***p < 0.001 (Student *t*‐test).

We first evaluated the biological effect of GP‐2250 (Figure 1) on the panel of 10 ovarian cancer cell lines using cell viability assay. The half‐maximal inhibitory concentration (IC_50_) of GP‐2250 in ovarian cancer cells ranged from 3.8 ± 0.2 to 289.8 ± 5.4 μmol/L after 72 h of treatment. As shown in Figure 2, Kuramochi, OVCAR4, and OVCAR8 cells were more sensitive to GP‐2250 (<100 μmol/L IC_50_) than were A2780, Caov3, OVCAR3, OVCAR5, HeyA8, HeyA8‐MDR, and SKOV3 cells (>200 μmol/L IC_50_). We confirmed that GP‐2250 reduced the proliferation and increased the apoptosis of ovarian cancer cells (Figure S1). Also, we found that HRD positive ovarian cancer cells (Kuramochi, OVCAR4, and OVCAR8) were more vulnerable to GP‐2250 than HRP ovarian cancer cells (A2780 and OVCAR5). Thus, we chose Kuramochi, OVCAR4, and OVCAR8 cells for further studies. A previous study revealed that GP‐2250 alone had dose‐dependent cytotoxic effects and synergized with gemcitabine in pancreatic adenocarcinoma models.^15^, ^19^ To determine the biological effects of GP‐2250 in combination with standard chemotherapy drugs on ovarian cancer cells, we tested GP‐2250 combined with paclitaxel, cisplatin, topotecan, or olaparib. We first assessed the cytotoxicity of these drugs individually and in combination with GP‐2250. Among all combinations, olaparib showed the most potent synergistic effect with GP‐2250 in OVCAR4 cancer cells, and GP‐2250 combination with paclitaxel or topotecan showed less synergistic effect than GP‐2250 and olaparib combination. (Figure 3A). Notably, we found a synergistic effect of GP‐2250 with olaparib in both HRD‐positive and HRD‐negative ovarian cancer cells (Figure 3B). GP‐2250 combined with the other two PARP inhibitors (niraparib and rucaparib) had similar synergy. It clearly showed that GP‐2250 enhanced the PARP inhibitor activity. However, among the PARP inhibitors alone, rucaparib has less effect than olaparib and niraparib (Figure S2). In addition, we observed a marked reduction in the number of OVCAR4 and OVCAR8 colonies that formed following treatment with GP‐2250, PARP inhibitors, and in combinations (Figure 3C).

### 3.2 GP‐2250 decreases the AKT/mTOR signaling pathway in ovarian cancer cells

**FIGURE 4:**
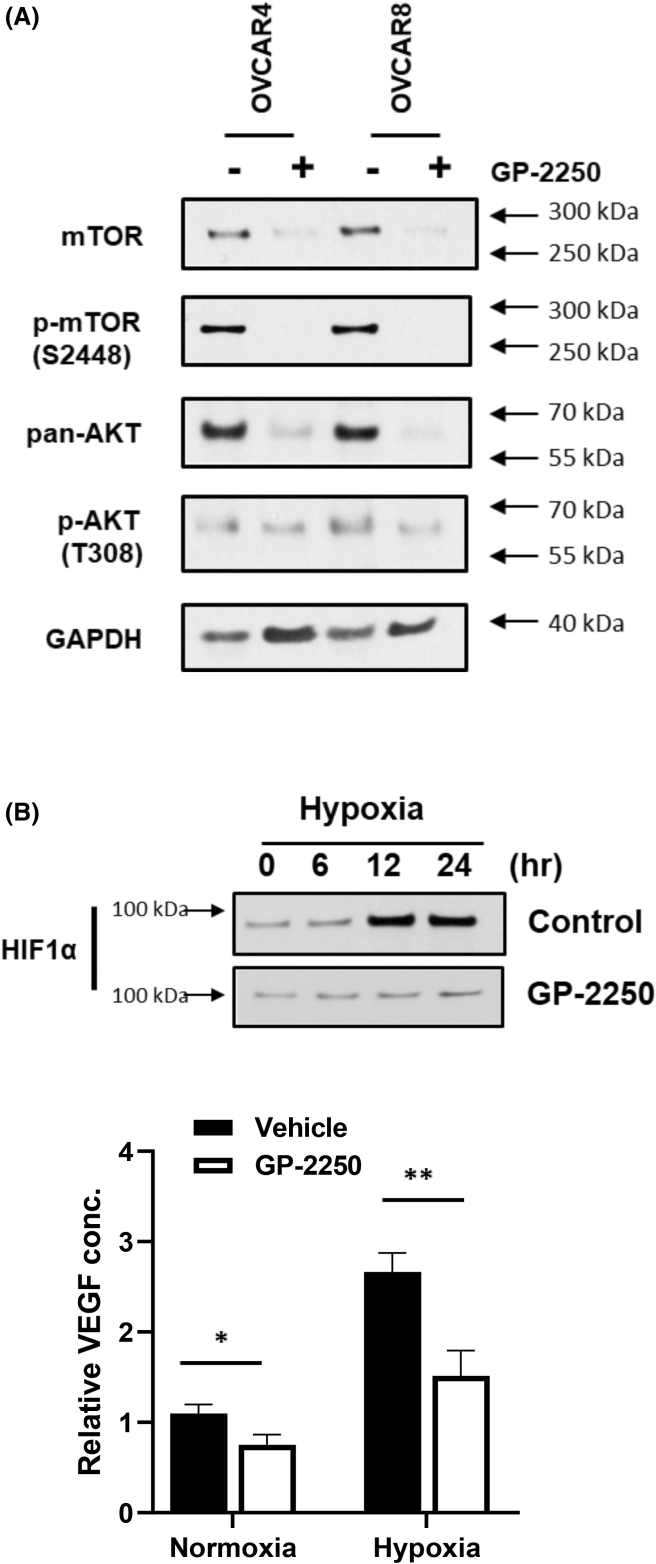
GP‐2250 inhibits mTOR, AKT, and HIF‐1α expression. (A) Ovarian cancer cells were treated with GP‐2250 for 24 h, and cell lysates were analyzed using Western blotting. (B) OVCAR8 cells were pretreated with a vehicle or GP‐2250 for 6 h and incubated in a hypoxia incubator (1% oxygen) for the indicated times. HIF‐1α expression in the cells was measured using Western blotting (top). An enzyme‐linked immunosorbent assay was used to determine VEGF secretion levels in the cells (bottom). The error bars indicate mean (± SD) values and are representative of three biological experiments. *ns*, not significant, *p < 0.05; **p < 0.01 (vs. control; Student *t*‐test).

Next, we performed RPPA analysis to identify the downstream pathways that are impacted by GP‐2250 treatment. We treated OVCAR4 and OVCAR8 cells with a vehicle (control) or GP‐2250 for 6 and 24 h and then subjected cell lysates to RPPA analysis. Ingenuity Pathway Analysis identified that GP‐2250 inhibited AKT and mTOR kinase activation and expression (data not shown). Western blot analysis confirmed decreased phospho‐AKT, AKT, phospho‐mTOR, and mTOR levels in these cells (Figure 4A). In addition, RPPA results revealed that GP‐2250 decreased hypoxia‐inducible factor (HIF)‐1α expression level. As shown in Figure 4B, HIF‐1α expression level was increased 6 h after exposure to hypoxic conditions (1% oxygen), reaching a maximum level in 12 h. However, HIF‐1α expression level did not increase under hypoxic conditions after pretreatment with GP‐2250 for 2 h. Given the role of HIF‐1α in VEGF regulation,^20^ we further examined the effects of GP‐2250 on VEGF secretion. Hypoxia increased VEGF secretion 2.4‐fold over that under normoxic conditions. However, treatment with GP‐2250 significantly abrogated VEGF secretion.

### 3.3 GP‐2250 decreases cell glycolysis through the inhibition of HK activity

**FIGURE 5:**
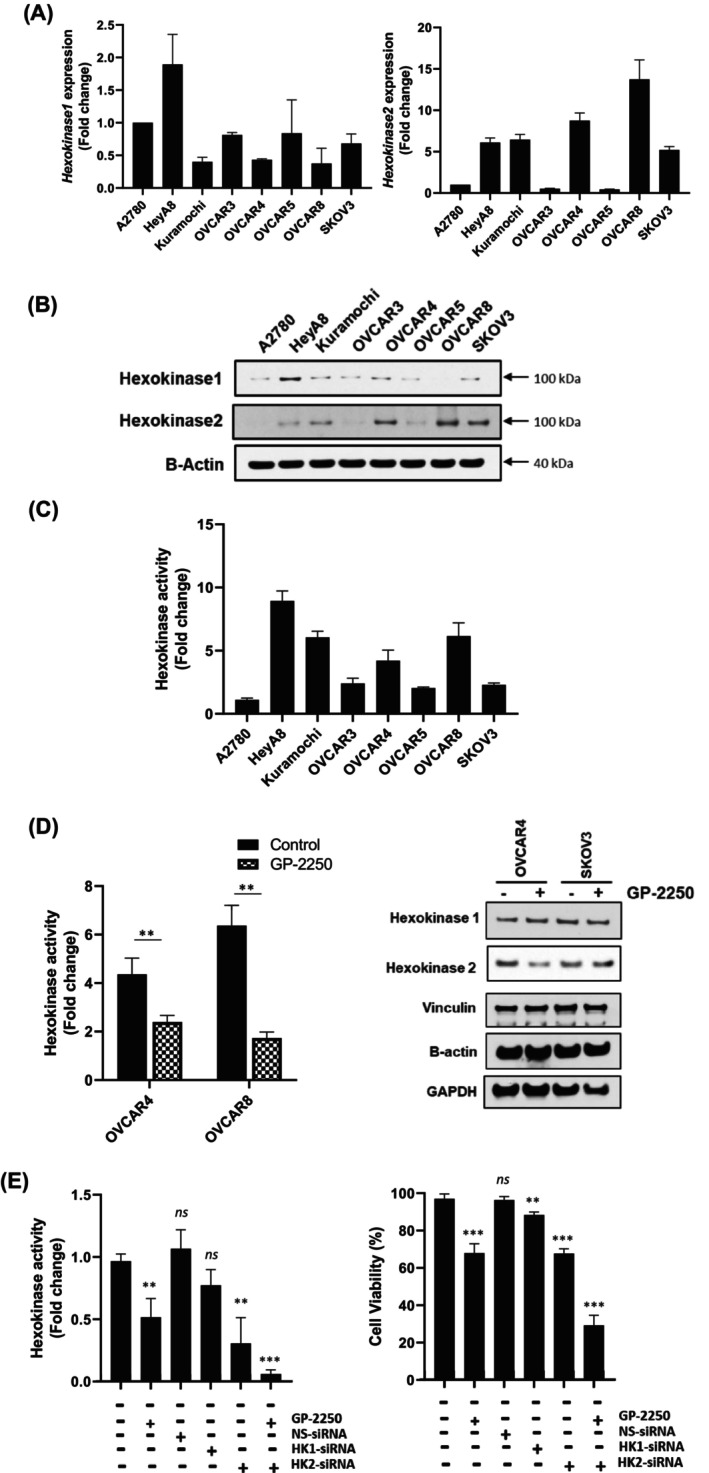
GP‐2250 decreases glycolysis via inhibition of HK2 activation and expression. HK1 and HK2 mRNA (A) and protein (B) expression levels in ovarian cancer cells were determined. (C) The indicated ovarian cancer cell lysates were prepared, and the HK activity in them was measured according to NADH level. (D) Ovarian cancer cells were pretreated with GP‐2250 for 6 h and then cultured for 24 h. The HK activity and protein expression levels in the cells were then determined. (E) OVCAR8 cells were transiently transfected with siRNA targeting HK1 or HK2 following treatment with or without GP‐2250 and then further cultured for 24 h (Hexokinase activity) or 72 h (Cell viability). The error bars indicate mean (± SD) values and are representative of three biological experiments. **p < 0.01, ***p < 0.001 (vs. control; Student *t*‐test).

Next, we investigated the metabolic changes in cancer cells treated with GP‐2250 using ultrahigh‐resolution mass spectrometry. We treated ovarian cancer cells with GP‐2250 for 8, 24, and 48 h and then prepared metabolite extracts as described in Section 2. We analyzed the data using TraceFinder software and normalized the DNA concentrations according to each metabolite's relative abundance. Metabolomic analysis revealed a reduction in the level of glucose‐6‐phosphate and fructose‐6‐phosphate, which are upstream metabolites of glycolysis. We also observed that treatment with GP‐2250 reduced pyruvate and ATP production and increased cellular ROS levels in a dose‐dependent manner in ovarian cancer cells (Figure S3).^15^ An important hallmark of many cancers is the need to metabolize glucose at an elevated rate. HK plays a critical role in glucose metabolism to produce glucose‐6‐phosphate, the first intermediate of glycolysis and the pentose phosphate pathway, and plays a central role in energy metabolism.^21^, ^22^ To determine whether treatment with GP‐2250 affects upstream metabolites of glycolysis, we examined HK1 and HK2 mRNA and protein expression and activity levels in ovarian cancer cell lines (Figure 5A–C). We found various expressions of HK1 and HK2 mRNA and protein levels in ovarian cancer cells. Interestingly, we found that high expression of HK2 but not HK1 correlated with GP‐2250 sensitivity. SKOV3 was not vulnerable to GP‐2250 even though it showed a high level of HK2. Next, we examined the effects of GP‐2250 on HK2 activity and expression. We treated OVCAR4 and OVCAR8 cells with a vehicle or GP‐2250 for 12 h and then determined HK activity and protein expression in cell lysates. HK activity was significantly decreased by GP‐2250 exposure in both OVCAR4 and OVCAR8 cells. Western blot analysis demonstrated that HK2 protein expression was reduced following treatment with GP‐2250; however, HK1 expression was not affected (Figure 5D). We confirmed these results by employing transient transfection of siRNA targeting HK1 and HK2. As shown in Figure 5E, we found a significant reduction of HK activity and cell viability induced by treatment with GP‐2250 alone or along with siRNA targeting HK2 rather than HK1. In addition, we found a more robust decrease in HK activity and cell viability when we combined the HK2 siRNA with GP‐2250 than when we combined HK1 siRNA with GP‐2250. These results indicated that the inhibition of expression and activity of HK2 by GP‐2250 decreases cell glycolysis and increases ROS level (Figure S3); as a result, GP‐2250 increases anti‐neoplastic effects on cancer cells. siRNA sequences targeting HK1 and HK2 were validated by downregulating their protein expression level using Western blot analysis (Figure S4).

### 3.4 GP‐2250 is highly effective in combination with PARP inhibitor and bevacizumab‐based therapy

**FIGURE 6:**
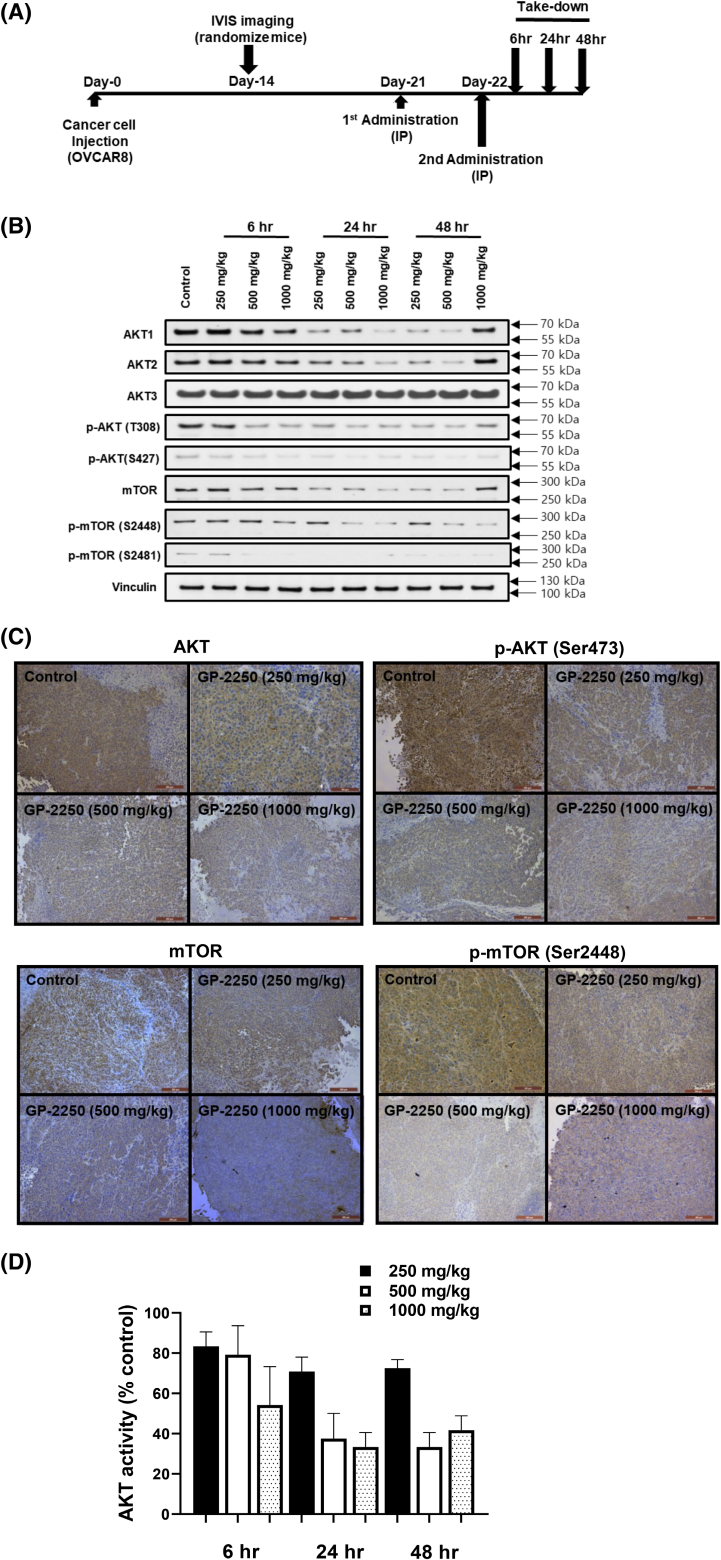
Determination of the most therapeutically effective GP‐2250 dose in vivo. (A) Schematic of in vivo PD study of GP‐2250 in the OVCAR8 mouse model. (B) Western blot of the total cell lysates from mouse tumor samples at the indicated takedown times using the indicated antibodies. (C) Immunohistochemical stains of paraffin slides for the expression of AKT, phospho‐AKT, mTOR, and phospho‐mTOR. Three slides with five fields per slide were examined. (D) AKT kinase activity in tumor tissue. AKT activity in ovarian tumor samples was measured at the indicated takedown times.

To determine the effective dose and interval of GP‐2250 treatment, we performed an in vivo PD study with an orthotopic luciferase‐labeled OVCAR8 mouse model of ovarian cancer. In this study, we randomized mice after confirmation of tumor establishment via bioluminescent imaging on Day 14 and gave three dose levels of GP‐2250 (250, 500, and 1000 mg/kg) two times on Days 21 and 22 with 24‐h intervals. We then euthanized the mice 6, 24, and 48 h after treatment (Figure 6A). As expected, the mouse body and tumor weights did not differ in each group (Figure S6). We assessed the PD response using AKT kinase assay, Western blot analysis, and immunohistochemical analysis with anti‐phospho‐AKT, anti‐AKT, anti‐mTOR, and anti‐phospho‐mTOR antibodies; we selected these markers based on the RPPA results. As shown in Figure 6B, among the mice given 500 or 1000 mg/kg GP‐2250, the phosphorylation and expression of mTOR decreased after 24 h of treatment. AKT1, AKT2, and mTOR protein levels were also decreased at 24 h of treatment with 500 or 1000 mg/kg GP‐2250. The decreased total and phosphorylation level of these proteins was sustained until 48 h of treatment; however, the phosphorylation and expression of AKT3 did not change. We also performed immunohistochemical staining of ovarian tumors for AKT and mTOR of mouse tumor samples (Figure 6C). To further assess the effect of GP‐2250, we measured AKT kinase activity in tumor samples. We found that 500 and 1000 mg/kg GP‐2250 treatment was the most effective treatment for AKT inhibition (Figure 6D). Based on these results, we administered a 500 mg/kg GP‐2250, twice weekly in subsequent therapy experiments.

### 3.5 Combination of GP‐2250 with PARP inhibitors

**FIGURE 7:**
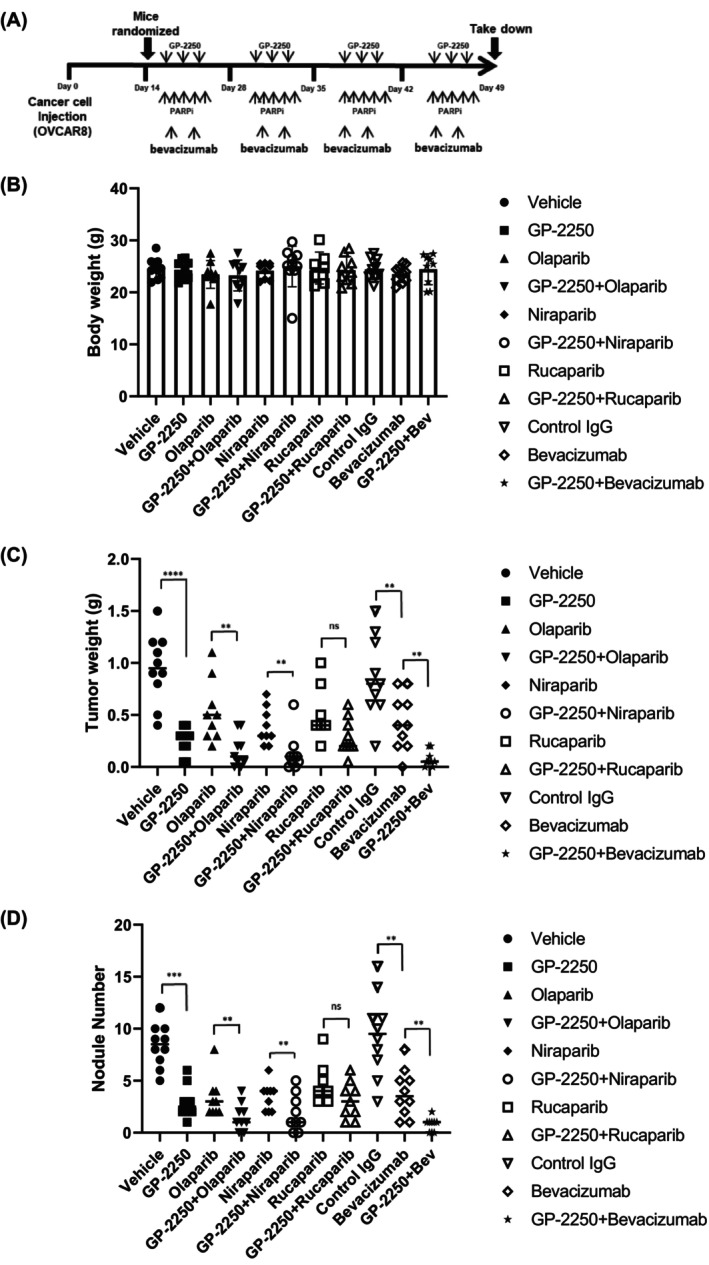
Antitumor effect of GP‐2250 combined with olaparib or bevacizumab in the OVCAR8 mouse model. (A) Schematic of the experimental protocol. (B–D) Body weights (B), tumor weights (C), and nodule numbers (D) for the OVCAR8 mouse model after treatment with a vehicle or normal IgG (control), GP‐2250, PARP inhibitors (olaparib, niraparib, and rucaparib), bevacizumab (Bev), or combinations of GP‐2250 with PARP inhibitors or bevacizumab. The error bars indicate mean (± SD) values compared with the control group using the Student *t*‐test and are representative of three biological experiments. *ns*, not significant, *p < 0.05, **p < 0.01; ***p < 0.001; ****, *p* < 0.0001 (Student *t*‐test).

To evaluate the therapeutic efficacy of GP‐2250 alone and in combination with PARP inhibitors, we used the OVCAR8 orthotopic ovarian cancer model. We randomized female tumor‐bearing nude mice into eight treatment groups (10 mice/group): vehicle (control), 500 mg/kg GP‐2250, 50 mg/kg olaparib, 50 mg/kg niraparib, 50 mg/kg rucaparib, GP‐2250 combined with olaparib, GP‐2250 combined with niraparib, and GP‐2250 combined with rucaparib. We started treatment 2 weeks after tumor cell injection (Figure 7A). The mice were treated with intraperitoneal administration of GP‐2250 twice a week and oral administration of PARP inhibitors daily as described previously.^23^ At the end of the experiment, on Day 49, the mouse body weights in all groups did not differ markedly (Figure 7B). As shown in Figure 7C and D, the mean (± SD) tumor weights in the GP‐2250 (0.25 ± 0.12 g; *p* < 0.001), olaparib (0.53 ± 0.29 g; *p* < 0.05), niraparib (0.38 ± 0.17 g; *p* < 0.001), and rucaparib (0.52 ± 0.27 g; *p* < 0.05) groups were significantly lower than that in the control group (0.95 ± 0.33 g). In combination treatment, we observed a significant reduction in tumor weight in the GP‐2250 combination with olaparib (0.16 ± 0.15 g; *p* < 0.05) and niraparib (0.13 ± 0.18 g; *p* < 0.05) groups compared to those of PARP inhibitor monotherapy group; olaparib (0.53 ± 0.29 g) and niraparib (3.09 ± 0.17 g). However, the combination of GP‐2250 and rucaparib (0.29 ± 0.17 g; *p* < 0.05) did not show significant tumor reduction compared to those of rucaparib monotherapy treatment group (0.53 ± 0.27 g). Also, tumor nodule number was significantly lower in the GP‐2250 (2.90 ± 1.52; *p* < 0.001), olaparib (3.30 ± 1.93; *p* < 0.001), niraparib (3.40 ± 0.44; *p* < 0.001), and rucaparib (4.80 ± 2.11; *p* < 0.01) groups than in the control group (8.40 ± 2.06). The relative reductions in tumor nodule number in monotherapy groups were 65.4% in the GP‐2250 group, 60.7% in the olaparib group, 59.5% in the niraparib group, and 42.8% in the rucaparib group, respectively. A significant reduction of tumor nodule number was observed in the GP‐2250 combination with olaparib compared to those of olaparib monotherapy group. These results demonstrated that mice given GP‐2250 combinations of olaparib had substantially lower tumor weights and fewer nodules than those in the monotherapy groups.

### 3.6 Combination of GP‐2250 with bevacizumab

Given the observed effects of GP‐2250 on HIF‐1α expression, we next examined the combination of GP‐2250 and bevacizumab in vivo. Specifically, we subjected the OVCAR8 xenograft mouse model to treatment with normal IgG (control; 6.25 mg/kg twice a week), GP‐2250, bevacizumab (6.25 mg/kg twice a week), or GP‐2250 combination with bevacizumab intraperitoneally. As shown in Figure 7C,D, mouse body weights did not change significantly with treatment. We observed that the mean (± SD) tumor weights in the GP‐2250 (0.25 ± 0.12 g; *p* < 0.001), bevacizumab (0.43 ± 0.26 g; *p* < 0.05), and GP‐2250 plus bevacizumab (0.07 ± 0.07 g; *p* < 0.001) groups were significantly lower than that in control IgG group (0.86 ± 0.38 g). We also observed tumor nodule numbers in the bevacizumab (3.80 ± 2.25; *p* < 0.001) and GP‐2250 combination bevacizumab (0.77 ± 0.66; *p* < 0.0001) groups were lower than that in the normal IgG group (9.40 ± 3.92). We found that the GP‐2250 combination of bevacizumab showed the most profound antitumor effect.

## 4 DISCUSSION

**FIGURE 8:**
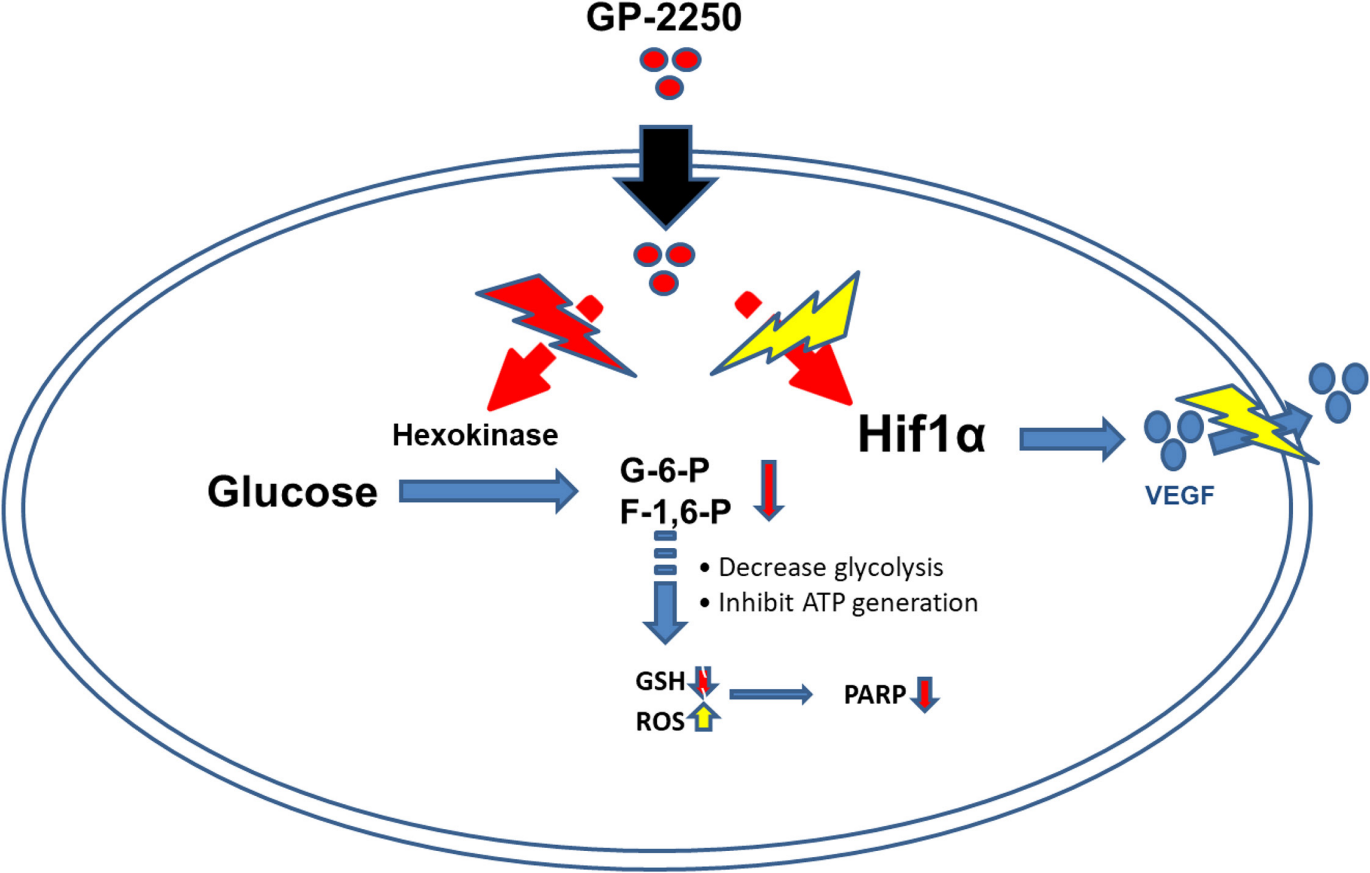
Schematic of the proposed biological effects of GP‐2250.

The key finding of our investigation is that GP‐2250 exerts a profound antitumor effect in combination with PARP inhibitors and bevacizumab. GP‐2250 is a novel cancer metabolism–based therapeutic agent currently in phase 1 clinical trials for advanced pancreatic cancer (NCT0384110) and should be extended to other types of cancer. Previous studies demonstrated that GP‐2250 has cytotoxic and antiproliferative effects on pancreatic carcinoma cells through oxidative‐stress–driven programmed cell death mechanism in vitro. Also, treatment with GP‐2250 reduced tumor growth both alone and, more significantly, combined with gemcitabine in vivo, with good tolerance and no secondary resistance.^10^, ^15^, ^19^ In the present study, we investigated the antitumor effect of GP‐2250 on various ovarian cancer cell lines and its therapeutic efficacy using an orthotopic ovarian cancer mouse model. The key finding of our investigation is that GP‐2250 inhibits ovarian cancer cell proliferation in vitro and has enhanced antitumor effects in combination with PARP inhibitors or bevacizumab. Physicians have mainly used PARP inhibitors such as olaparib in the maintenance treatment of patients with BRCA‐mutated or HRD positive ovarian cancer after responding to platinum‐based regimens.^24^, ^25^, ^26^ Furthermore, it has been reported that there is a significant benefit in patients with non‐BRCA‐mutated, platinum‐sensitive ovarian cancer and even lower in small numbers of those with platinum‐resistant ovarian cancer.^27^, ^28^ In the present study, we first investigated GP‐2250's direct inhibitory effect on ovarian cancer cell proliferation in a series of in vitro experiments, including MTT and BrdU assays, trypan blue exclusion, and an annexin V/propidium iodide (PI) assay. We found that HRD positive ovarian cancer cells (Kuramochi, OVCAR4, and OVCAR8) were more vulnerable to GP‐2250 than were HRP cells (A2780 and OVCAR5). Also, an in vitro study revealed that combinations of GP‐2250 with PARP inhibitors had greater synergistic effects than those of GP‐2250 with other standard‐of‐care drugs, such as paclitaxel, cisplatin, and topotecan. Further analysis showed a significant reduction of tumor weight in GP‐2250 combination with olaparib and niraparib, while a combination of GP‐2250 and niraparib was not significant. Additionally, we found synergistic effects of GP‐2250 combined with PARP inhibitors in HRD positive ovarian cancer cells. These results demonstrated that GP‐2250 might enhance the inhibition of DNA single‐strand break repair or promote double‐strand breaks, which can lead to cell death when the homologous recombination repair machinery is absent or compromised. Next, we investigated the molecular mechanism of GP‐2250 in ovarian cancer cells. We observed that GP‐2250 decreased the initial metabolites of glucose, such as glucose‐6‐phosphate, and GP‐2250 reduced HK2 expression and activity. In addition, HK2‐knockdown cells were more sensitive to the combination of GP‐2250 than nonspecific siRNA transfected control cells. Early studies demonstrated that a key hallmark of many cancers is the need to metabolize glucose at an elevated rate. HK plays a critical role in glucose metabolism to produce glucose‐6‐phosphate, the first intermediate of glycolysis and the pentose phosphate pathway that plays a central role in energy metabolism.^21^, ^22^ Among the HK isoforms, HK2, the most active isozyme, is markedly expressed in cancer cells, and its expression is related to the progression of and poor prognosis for glioblastoma and epithelial ovarian cancer.^29^, ^30^, ^31^ Also, Kaplan–Meier analysis demonstrated that increased HK2 expression was related to shortened progression‐free survival of patients with ovarian cancer (Figure S5). We demonstrated that GP‐2250 inhibits HK2, the rate‐limiting first step of glycolysis, resulting in decreased glycolysis. Consequently, it causes depleted ATP levels and increases ROS, which induces cell death.^15^, ^19^ In addition, it has been previously demonstrated that HK binds to PARP and is involved in DNA repair.^32^ The definitive mechanism behind GP‐2250 regulation of HK2 remains to be investigated. Our RPPA analysis revealed that the expression and activation of AKT and mTOR levels are decreased with GP‐2250, demonstrating that this may be a predicted biomarker of response to GP‐2250 treatment. We found that 500 mg/kg is the therapeutically effective dose of GP‐2250 because this dose most effectively reduced AKT and mTOR expression and activation. In a pancreatic cancer mouse model, GP‐2250 was safe and well‐tolerated, with no major organ toxicity.^13^, ^15^ Likewise, the mice given GP‐2250 in our in vivo studies had no weight loss or behavioral changes, a finding consistent with the absence of side effects of GP‐2250 at the therapeutically effective dose of 500 mg/kg. We subsequently noted a profound antitumor effect of GP‐2250 in the OVCAR8 mouse model and further demonstrated the antitumor efficacy of GP‐2250 when given with PARP inhibitors or bevacizumab. Of note, the combination of GP‐2250 and bevacizumab produced greater tumor reduction than each monotherapy and the combination of GP‐2250 with PARP inhibitors. We also found inhibition of HIF‐1α protein expression and VEGF secretion in cancer cells following treatment with GP‐2250 in vitro. These results support the therapeutic benefit of GP‐2250 in combination with bevacizumab in the mouse model of ovarian cancer. Anti‐VEGF therapy has been used in both up‐front and recurrent ovarian cancer patients: however, all tumors eventually develop resistance to anti‐VEGF, and the patient eventually succumbs to their disease.^33^ Even though this study showed a profound antitumor effect of GP‐2250 in combination with bevacizumab, further investigations are needed to evaluate GP‐2250 and bevacizumab combination effect on the adaptive resistance ovarian cancer model. In conclusion, we suggest that the mechanism behind GP‐2250's anti‐neoplastic effect on ovarian cancer cells includes inhibition of glycolysis via modulation of HK2 activation and expression and inhibition of HIF‐1α induced VEGF secretion. (Figure 8). Taken together, our in vivo results demonstrated that GP‐2250 combination with PARP inhibitors or bevacizumab is well tolerated and suggests a rational combination for further development.

## 5 CONCLUSIONS

GP‐2250 displayed anti‐neoplastic potential in pancreatic carcinoma cells; however, the inhibitory mechanism of antitumor effects and in vivo therapeutic efficacy have not been fully delineated. We exploited the mechanism of action and biological effects of GP‐2250 using in vitro and in vivo models of ovarian cancer, and results showed GP‐2250 exerts profound effects on tumor metabolism and, in combination with PARP inhibitors or bevacizumab therapy, provides a potential combination therapy for homologous recombination‐proficient ovarian cancers.

## AUTHOR CONTRIBUTIONS

**Mark S. Kim:** Conceptualization (lead); investigation (lead); methodology (equal); writing – original draft (lead). **Deanna Glassman:** Formal analysis (equal); investigation (equal); writing – review and editing (equal). **Katelyn F. Handley:** Investigation (equal); validation (equal); writing – review and editing (equal). **Adrian Lankenau Ahumada:** Writing – review and editing (equal). **Nicholas B. Jennings:** Project administration (equal); validation (equal); writing – review and editing (equal). **Emine Bayraktar:** Formal analysis (equal); writing – review and editing (equal). **Katherine Foster:** Data curation (equal); formal analysis (equal); investigation (equal); writing – review and editing (equal). **Robiya Joseph:** Methodology (equal); validation (equal); writing – review and editing (equal). **Sanghoon Lee:** Validation (equal); writing – review and editing (equal). **Robert L. Coleman:** Conceptualization (equal); writing – review and editing (equal). **Anil K. Sood:** Conceptualization (equal); funding acquisition (equal); project administration (equal); supervision (lead); writing – review and editing (equal).

## FUNDING INFORMATION

This study was supported by CPRIT grant PR 180381; National Cancer Institute grants award numbers: CA016672, CA227622 (AKS), CA177909 (AKS), CA209904 (AKS), MD Anderson Ovarian Cancer Moon Shot (AKS), SPORE in Ovarian Cancer CA281701 (AKS), T32 training grant CA101642 (DG), and CPRIT Grant No.: RP170593 (KFH), and also, supported, in part, by Panavance Therapeutics LS2019‐13035043‐GS (MSK).

## CONFLICT OF INTEREST STATEMENT

AKS reports consulting for Merck, AstraZeneca, Onxeo, ImmunoGen, Ivlon, GSK, and Kiyatec; being a stockholder in Bio‐Path Holdings. RLC reports consulting for Agenus, Alkermes, AstraZeneca, Clovis, Deciphera, Genelux, Genmab, GSK, Immunogen, OncoQuest, Onxerna, Regeneron, Roche/Genentech, Novocure, Merck, Abbvie and receiving research funding from AstraZeneca, Clovis, Genelux, Genmab, Merck, Immunogen, Roche/Genentech. MSK received research support from Panavance Therapeutics. All other authors declare no potential conflicts of interest.

## Supporting information


Figure S1.

Table S1.

## Data Availability

Data supporting the findings of this study are available within the paper and its Supplementary files. A data use agreement will be required before review board approval, as appropriate.
