# Supplementary material for: Mechanism and rational combinations with GP‐2250, a novel oxathiazine derivative, in ovarian cancer
Source: Cancer Med. 2024 Aug 8;13(15):e70031. doi: 10.1002/cam4.70031 (PMC11306972; doi:10.1002/cam4.70031)
Supplement: Supplementary file 1 — Figure S1. [file CAM4-13-e70031-s001.pdf]

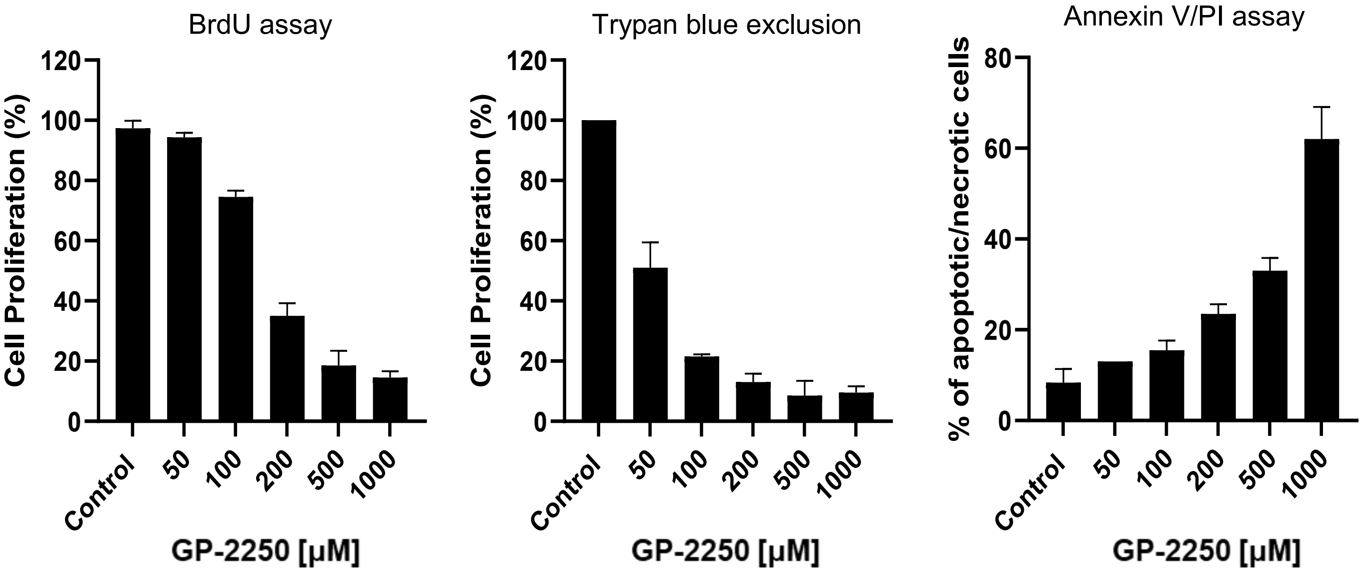

Supplementary Fig. 1. Effect of GP-2250 on OVCAR8 cells. Cells were treated indicated concentration of GP-2250 and the cell viability was analyzed (A) BrdU assay, (B) Trypan blue exclusion and (C) Annexin V/PI staining.

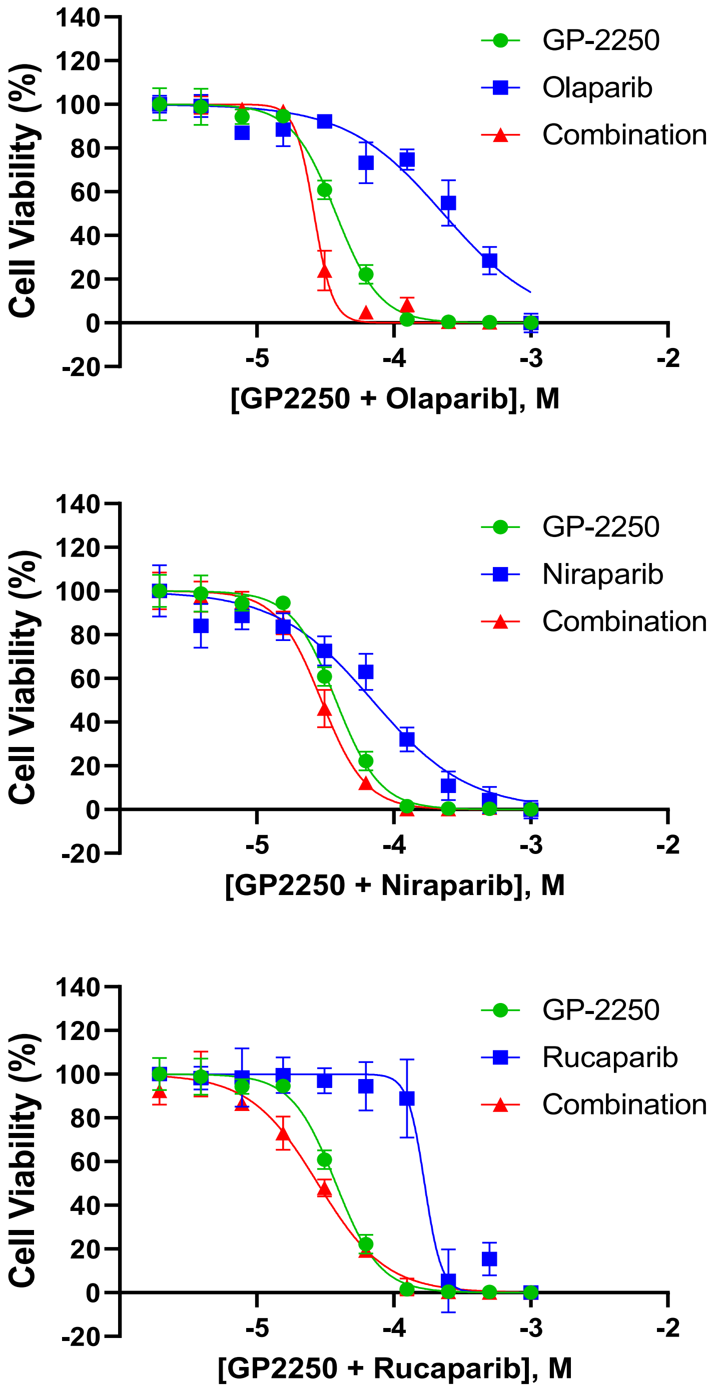

Supplementary Fig. 2. Effect of GP-2250 combination with PARP inhibitors on ovarian cancer cells. Cell viability assay. Cancer cells were treated with GP-2250 and indicated PARP inhibitors alone and combined with the indicated concentrations for 72 hrs. Cell viability was analyzed using CellTiter Glo assay.

Supplementary Fig. 3

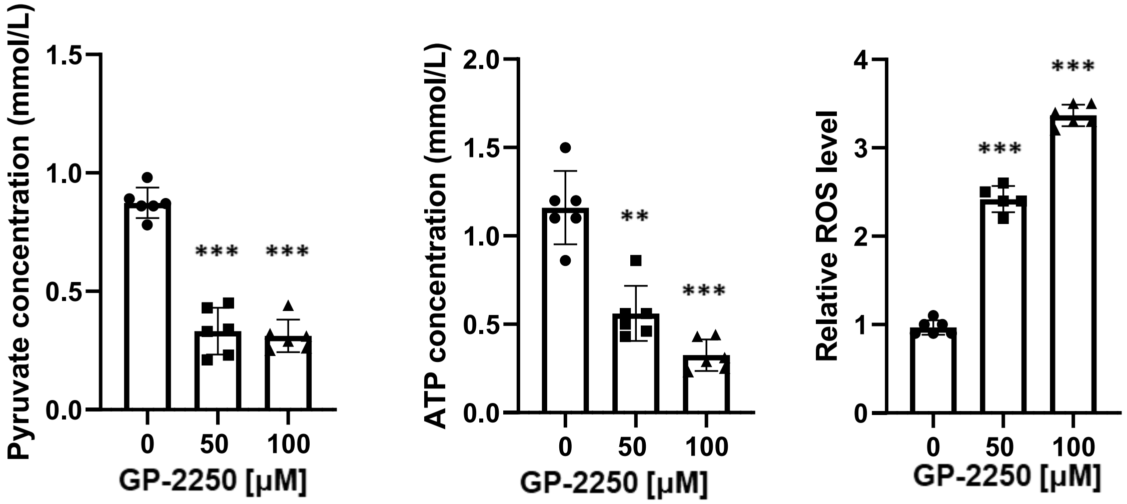

Supplementary Fig. 3. Impact of GP-2250 on glycolysis, ATP, and reactive oxygen species (ROS) level. OVCAR8 cells were treated with an indicated concentration of GP-2250, and glycolysis, ATP, and Ros level were analyzed.

Supplementary Fig. 4

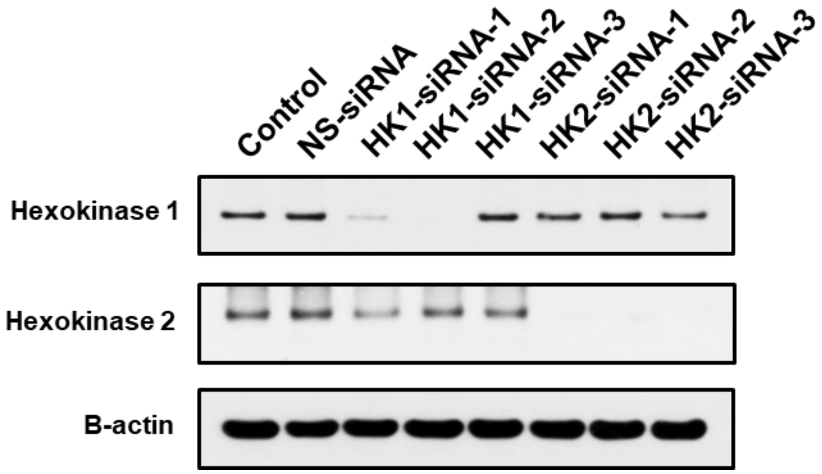

Supplementary Fig. 4. Determine HK1 and HK2 expression levels using various siRNAs targeting HK1 and HK2.

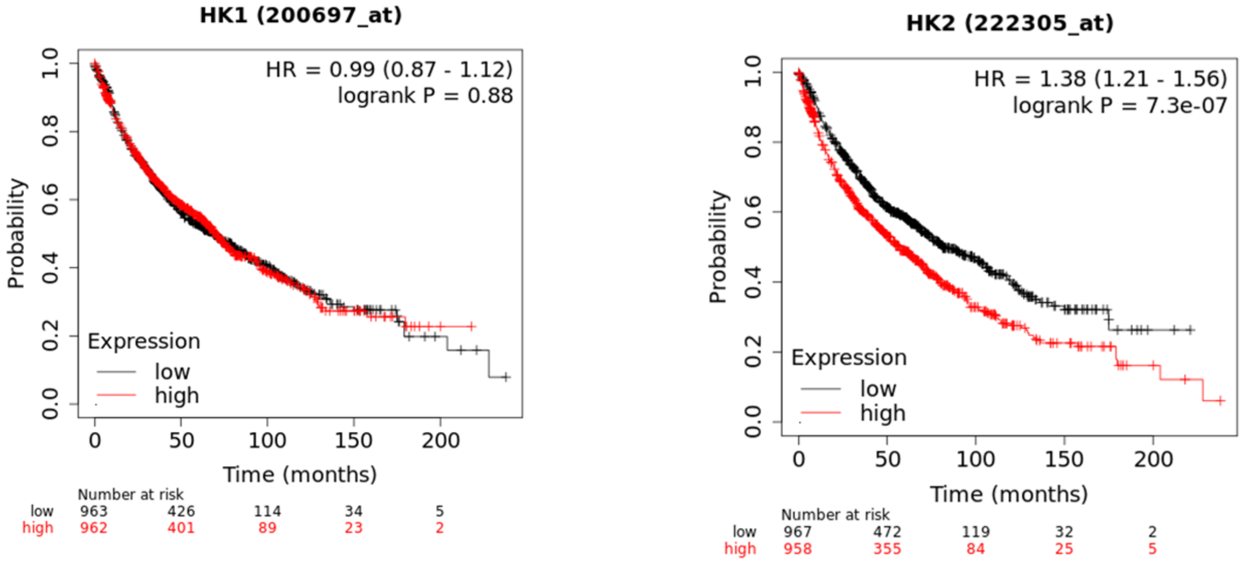

Supplementary Fig. 5. Kaplan-Meier survival curves of ovarian cancer patients by HK1 and HK2 level.

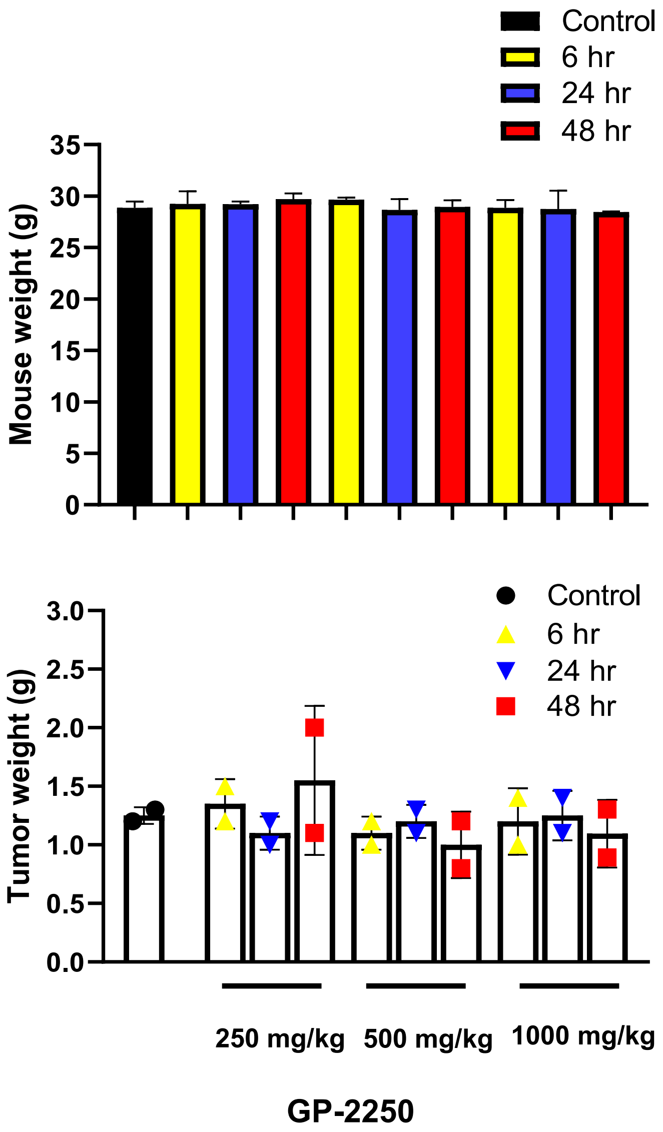

Supplementary Fig. 6. Determination of effective dose of GP-2250 in OVCAR8 mouse model. (A) Mouse weight. (B) Tumor weight.
