# Supplementary material for: Mechanism and rational combinations with GP‐2250, a novel oxathiazine derivative, in ovarian cancer
Source: Cancer Med. 2024 Aug 8;13(15):e70031. doi: 10.1002/cam4.70031 (PMC11306972; doi:10.1002/cam4.70031)
Supplement: Supplementary file 2 — Table S1. [file CAM4-13-e70031-s002.docx]

**Supplementary Table S1.** siRNA sequences for *HK1* and *HK2*.

| **siRNA Name** | **Sequence (5’ – 3’)** |
| --- | --- |
| HK1-1 | CUCUUCGAGCUGCACAACA[dT][dT]  UGUUGUGCAGCUCGAAGAG[dT][dT] |
| HK1-2 | CGCUCUUCGAGCUGCACAA[dT][dT]  UUGUGCAGCUCGAAGAGCG[dT][dT] |
| HK1-3 | CUGAUUCUGAGCGAUGAGA[dT][dT]  UCUCAUCGCUCAGAAUCAG[dT][dT] |
| HK2-1 | GAGUUGAUUCCACUAGUAU[dT][dT]  AUACUAGUGGAAUCAACUC[dT][dT] |
| HK2-2 | GUUAUGGGUACCUCUCUUA[dT][dT]  UAAGAGAGGUACCCAUAAC[dT][dT] |
| HK2-3 | GCAAGAUGUCCCAGUAAGA[dT][dT]  UCUUACUGGGACAUCUUGC[dT][dT] |
